# The Mechanism of TNF-*α*-Mediated Accumulation of Phosphorylated Tau Protein and Its Modulation by Propofol in Primary Mouse Hippocampal Neurons: Role of Mitophagy, NLRP3, and p62/Keap1/Nrf2 Pathway

**DOI:** 10.1155/2022/8661200

**Published:** 2022-08-12

**Authors:** Lin Zhang, Hong Song, Jie Ding, Dong-jie Wang, Shi-peng Zhu, Chi Liu, Xian Jin, Jia-wei Chen

**Affiliations:** ^1^Department of Anesthesiology, Jing'an District Central Hospital of Shanghai, Fudan University, No259 XiKang Road, Shanghai 200040, China; ^2^Department of Critical Care Medicine, Jing'an District Central Hospital of Shanghai, Fudan University, No259 XiKang Road, Shanghai 200040, China; ^3^Department of Anesthesiology, Fudan University Shanghai Cancer Center, Department of Oncology, Shanghai Medical College, Fudan University, No270 DongAn Road, Shanghai 200032, China; ^4^Department of Geriatrics Center, National Clinical Research Center for Aging and Medicine, Jing'an District Central Hospital of Shanghai, Fudan University, No259 XiKang Road, Shanghai 200040, China

## Abstract

**Background:**

Neuroinflammation-induced phosphorylated Tau (p-Tau) deposition in central nervous system contributes to neurodegenerative disorders. Propofol possesses neuroprotective properties. We investigated its impacts on tumor necrosis factor-*α* (TNF-*α*)-mediated p-Tau deposition in neurons.

**Methods:**

Mouse hippocampal neurons were exposed to propofol followed by TNF-*α*. Cell viability, p-Tau, mitophagy, reactive oxygen species (ROS), NOD-like receptor protein 3 (NLRP3), antioxidant enzymes, and p62/Keap1/Nrf2 pathway were investigated.

**Results:**

TNF-*α* promoted p-Tau accumulation in a concentration- and time-dependent manner. TNF-*α* (20 ng/mL, 4 h) inhibited mitophagy while increased ROS accumulation and NLRP3 activation. It also induced glycogen synthase kinase-3*β* (GSK3*β*) while inhibited protein phosphatase 2A (PP2A) phosphorylation. All these effects were attenuated by 25 *μ*M propofol. In addition, TNF-*α*-induced p-Tau accumulation was attenuated by ROS scavenger, NLRP3 inhibitor, GSK3*β* inhibitor, or PP2A activator. Besides, compared with control neurons, 100 *μ*M propofol decreased p-Tau accumulation. It also decreased ROS and NLRP3 activation, modulated GSK3*β*/PP2A phosphorylation, leaving mitophagy unchanged. Further, 100 *μ*M propofol induced p62 expression, reduced Keap1 expression, triggered the nuclear translocation of Nrf2, and upregulated superoxide dismutase (SOD) and heme oxygenase-1 (HO-1) expression, which was abolished by p62 knockdown, Keap1 overexpression, or Nrf2 inhibitor. Consistently, the inhibitory effect of 100 *μ*M propofol on ROS and p-Tau accumulation was mitigated by p62 knockdown, Keap1 overexpression, or Nrf2 inhibitor.

**Conclusions:**

In hippocampal neurons, TNF-*α* inhibited mitophagy, caused oxidative stress and NLRP3 activation, leading to GSK3*β*/PP2A-dependent Tau phosphorylation. Propofol may reduce p-Tau accumulation by reversing mitophagy and oxidative stress-related events. Besides, propofol may reduce p-Tau accumulation by modulating SOD and HO-1 expression through p62/Keap1/Nrf2 pathway.

## 1. Introduction

Neurodegenerative disorders, such as Parkinson's disease (PD), Alzheimer's disease (AD), and perioperative neurocognitive disorder (PND), are diseases in which the structure and function of neurons are impaired, leading to dysfunction of central nervous system (CNS). While the causes associated with neuronal impairment remain poorly understood, increasing evidence proved systemic inflammatory response especially neuroinflammation is crucial in the progression of neurodegenerative diseases [1, 2]. The core of neuroinflammation is likely the same in aging, metabolic diseases such as hypertension and diabetes, or cerebral insults such as stroke and injury [3], and inflammatory mediator tumor necrosis factor-*α* (TNF-*α*) was proved to serve as a key player and biomarker of neuroinflammation [4]. Speaking of molecular mechanisms, plenty of evidence suggested that the aggregation of *β*-amyloid, *α*-synuclein, and Tau protein plays a crucial role in neurodegenerative cascades [5–7]. However, tauopathy especially hyperphosphorylation of Tau, rather than Tau itself, was believed to lead to dementia and neurodegenerative diseases [8]. A recent clinical study also revealed a robust relationship between phosphorylated Tau protein (p-Tau) in the brain and the extent of neurodegeneration in AD patients [9]. Nevertheless, whether neuroinflammation caused p-Tau accumulation was still unknown, and if so, how neuroinflammation triggered p-Tau accumulation was also rarely studied. Although posttranslational Tau protein modifications may be mediated by many factors, mitochondrial autophagy (also known as mitophagy), reactive oxygen species (ROS), the activation of the NOD-like receptor protein 3 (NLRP3) inflammasome, and the regulation of kinases and phosphatases have attracted attention due to their upstream and downstream effects on tauopathy [10–12].

Propofol is widely used as an intravenous anesthetic/sedative agent in clinical practice. Apart from hypnotic advantages, it possesses anti-inflammation [13] and antioxidation effects [14] as well as neuro-protective properties [15]. On cellular and molecular levels, propofol has been shown to attenuate TNF-*α*-induced neuronal dysfunction [16, 17]. It has also been reported to diminish mitochondrial dysfunction and ROS production in isolated rat hippocampal neurons [18, 19]. In addition, animal studies revealed that propofol exerted cognitive protection by regulating the expression and posttranslational modification, mostly phosphorylation of Tau in rat models [20, 21]. Nevertheless, deeper investigation concerning how propofol modulates Tau protein phosphorylation needs to be carried out.

In the present *in vitro* study, we examined whether and how TNF-*α* caused p-Tau accumulation in hippocampal neurons. More importantly, we investigated the protective effects and mechanisms of propofol in neurons. The findings of this study may provide potential therapeutic targets for the prevention and treatment of p-Tau accumulation and resultant neuron injury as well as neurodegenerative disorders.

## 2. Materials and Methods

### 2.1. Experimental Design

To examine the effects of TNF-*α* on p-Tau accumulation, neurons were exposed to different concentrations of TNF-*α* (10, 20, 40, 80, and 160 ng/mL) for different durations (1, 2, 4, and 8 h). Neuron viability and the amount of Tau as well as p-Tau were examined, and the optimal TNF-*α* treatment condition was determined. To investigate the protective effects of propofol, neurons were incubated with different concentrations (1, 5, 10, 25, 50, and 100 *μ*M) of propofol for 1 h followed by TNF-*α* treatment. We intended to examine the effect of propofol on TNF-*α*-induced p-Tau accumulation, and further investigated the mechanisms including mitophagy, ROS, NLRP3 inflammasome, glycogen synthase kinase-3*β* (GSK3*β*), cAMP-dependent protein kinase (PKA), protein phosphatase 2A (PP2A), antioxidant enzyme superoxide dismutase (SOD), heme oxygenase-1 (HO-1), NADPH quinine oxidoreductase 1 (NQO1), and p62/Keap1/Nrf2 pathway. To confirm their roles, inhibitors and activators as well as overexpression/knockdown technique were applied.

### 2.2. Cell Culture

Cryopreserved primary mouse hippocampal neurons were commercially obtained from Gibco-Life Technologies (Carlsbad, CA, USA) and cultured in B-27 Plus Neuronal Culture System (Gibco-Life Technologies, Carlsbad, CA, USA). After thawed and seeded in tissue culture flasks containing 5 mL media supplemented with neuronal growth supplement, 1% penicillin/streptomycin and 5% fetal bovine serum, neurons were kept in a humidified incubator filled with 5% CO_2_ and 95% air at 37°C. The culture media were replaced every other day, until neurons reached about 70% confluency and were ready for experiments without subculturing.

### 2.3. TNF-*α* Treatment and Propofol Pretreatment Protocol

Recombinant mouse TNF-*α* was obtained from Sigma-Aldrich (St. Louis, MO, USA) and was reconstituted with sterile water to a stock concentration of 0.1 mg/mL. To investigate the effect of TNF-*α* on p-Tau accumulation, neurons were exposed to different concentrations of TNF-*α* (10, 20, 40, 80, and 160 ng/mL) for different durations (1, 2, 4, and 8 h). By measuring the expression and phosphorylation of Tau protein, we aimed to determine the optimal condition, under which TNF-*α* exerted significant effect on the accumulation of p-Tau.

To investigate the protective effects of propofol against TNF-*α* in hippocampal neurons, we incubated neurons with different concentrations (1, 5, 10, 25, 50, and 100 *μ*M) of propofol (Sigma-Aldrich, St Louis, MO, USA) or its solvent 0.1% dimethyl sulfoxide (DMSO, Sigma-Aldrich, St Louis, MO, USA) for 1 h followed by TNF-*α* treatment (with the presence of propofol or DMSO). By observing the expression and phosphorylation of Tau protein, we intended to identify the optimal concentration, at which propofol exerted protective effects against p-Tau accumulation.

### 2.4. Cell Viability Assay

3-4,5-dimethylthiazol-2,5-diphenyltetrazolium bromide (MTT) assay was used to assess the viability of neurons. Briefly, neurons were seeded in 6-well culture plates and exposed to respective treatment. After removing culture media, neurons were rinsed with phosphate-buffered saline (PBS). MTT was dissolved in serum-free medium at a final concentration of 0.5 mg/mL and each well was loaded with 150 *μ*L MTT. After incubating at 37°C for 30 min, 150 *μ*L dimethyl formamide was added, and the incubation continued for 4 h, during which formazan crystals formed. Then, 150 *μ*L DMSO was added to dissolve formazan crystals. A microplate reader (Bio-Rad, Hercules, CA, USA) was used to determine the absorbance values at 570 nm, and optical density served as unit. Cell viability was expressed as the percentage of absorbance of treated neurons compared with that of untreated control neurons.

### 2.5. Mitochondrial Membrane Potential (MMP) Determination

MMP was determined through fluorescent dye rhodamine-123 (Rh123), which is a lipophilic cationic fluorescent probe for mitochondria, and the fluorescence was examined through flow cytometry with the use of fluorescence-activated cell sorter (FACS). Briefly, Rh123 (Beyotime Institute of Biotechnology, Shanghai, China) was dissolved in DMSO to make a 5 mM stock solution. After treatment, neurons were washed with PBS and incubated with 5 *μ*M Rh123 at 37°C in a dark chamber for 30 min. Then, neurons were washed with PBS to remove excess dye, and BD FACSPresto™ System (BD Biosciences, San Jose, CA, USA) was applied to detect the fluorescent signal at an excitation wavelength of 490 nm and an emission wavelength of 585 nm. Data were expressed as mean ± standard deviation of fluorescent intensity of Rh123 staining.

### 2.6. Mitophagy Assessment

The extent of mitophagy was evaluated by using Mitophagy Detection Kit (Beyotime Institute of Biotechnology, Shanghai, China) according to the manufacturer's instructions. In brief, after treatment, neurons were washed with Hanks' HEPES solution, incubated with 0.1 *μ*M mitophagy dye working solution at 37°C for 30 min, and incubated with 1 *μ*M lysosome dye working solution at 37°C for 30 min. After washing with Hanks' HEPES solution to remove excessive dye, the fluorescence was detected at an excitation wavelength of 550 nm and an emission wavelength of 610 nm. Data were expressed as mean ± standard deviation of fluorescent intensity.

### 2.7. Intracellular ROS Measurement

Intracellular ROS was monitored using a ROS-sensitive fluorogenic dye. The method is based on fluorescent 2',7'-dichlorofluorescein (DCF), which is oxidatively converted from non-fluorescent 2',7'-dichlorodihydrofluorescein diacetate (DCFH-DA, Beyotime Institute of Biotechnology, Shanghai, China). In brief, neurons were seeded in 6-well culture plates and exposed to respective treatment. Thereafter, neurons were incubated with 10 *μ*M DCFH-DA for 30 min at 37°C. Then, the reaction mixture was aspirated and replaced with 200 *μ*L PBS in each well. The plates were placed on a shaker for 10 min at room temperature in the dark, and subject to fluorescence microplate reader with an excitation wavelength of 485 nm and an emission wavelength of 535 nm. The data were recorded as folds of increased fluorescence intensity in treated neurons compared with that of untreated neurons.

### 2.8. Mitochondrial ROS Assessment

ROS generation within mitochondrial compartment was assessed in live cells using MitoSOX Red, a fluorogenic dye that is taken up by mitochondria where it is readily oxidized by superoxide anion and serves as mitochondrial ROS indicator. Briefly, neurons were seeded in 6-well culture plates, and MitoSOX Red (Beyotime Institute of Biotechnology, Shanghai, China) was dissolved in DMSO to form 5 mM stock solution. After treatment, neurons were loaded with 1 *μ*M MitoSOX Red for 10 min at 37°C in the dark. Then, neurons were washed with PBS, and fluorescence intensity was determined with fluorescence microplate reader at 510 nm excitation and 580 nm emission, respectively. Data were recorded as folds of increased fluorescence intensity in treated neurons compared with that of untreated neurons.

### 2.9. Enzyme-Linked Immunosorbent Assay (ELISA)

The production of interleukin-1*β* (IL-l*β*) and interleukin-18 (IL-18) was evaluated by SimpleStep ELISA kit (Beyotime Institute of Biotechnology, Shanghai, China) according to the manufacturer's instructions. Briefly, after treatment, the culture media were harvested. In addition, neurons were scrapped off, suspended in PBS, and subject to ultrasonic homogenizer. Then, the homogenates and culture media were centrifuged at 2000 revolutions per minute (rpm) for 10 min at 4°C, and the supernatant was collected and transferred to 24-well plates precoated with antibody against IL-l*β* or IL-18. After incubating at 4°C for 30 min, the capture and detector antibody cocktail were added, and incubation lasted for 30 min at 4°C. Then, the supernatant was removed, and the wells were washed with PBS. Subsequently, the detection reagent was added, and the absorbance at 450 nm was measured with a microplate reader (Bio-Rad, Hercules, CA, USA). A standard curve was plotted using standard IL-1*β* or IL-18 supplied by the kit, and data were expressed as pg/mL.

### 2.10. Preparation of Whole Cell Extracts

After treatment, culture media were removed, and neurons were washed with PBS and then scraped off the culture flasks. After centrifugation for 5 min at 1000 rpm at 4°C, neuron pellets were suspended in radioimmunoprecipitation assay (RIPA) lysis solution (Santa Cruz Biotechnology, Santa Cruz, CA, USA) containing 1% protease inhibitor and 0.1% phosphatase inhibitor, and placed on ice for 10 min with intermittent homogenization by vortexing. Then, the whole cell proteins were obtained by centrifuging for 10 min at 5000 rpm, and the protein concentration was determined by BCA protein assay kit (Beyotime Institute of Biotechnology, Shanghai, China).

### 2.11. Preparation of Nuclear Extracts

Nuclear extracts were prepared using nuclear extract kit (Active Motif, Carlsbad, CA, USA) according to the manufacturer's protocol. After treatment, culture media were thoroughly removed. Then, neurons were washed with PBS, scraped off, transferred to prechilled tubes, pelleted by centrifugation at 1000 rpm for 5 min at 4°C, suspended in hypotonic buffer, and incubated on ice for 15 min. After adding detergent and intermittent vortexing for 10 sec, the suspensions were centrifuged at 14,000 rpm for 1 min at 4°C. Then, the pellets were suspended in complete lysis buffer, vortexed for 10 sec, and incubated for 30 min at 4°C. The suspensions were centrifuged at 14,000 rpm for 10 min at 4°C, and the supernatant was collected. Protein concentration was quantified by BCA protein assay kit (Beyotime Institute of Biotechnology, Shanghai, China), and the purity of nuclear fractions was verified by the absence of cytosolic marker *α*-tubulin.

### 2.12. Preparation of Cytosolic Extract

Cytosolic fractions were separated using ProteoExtract subcellular proteome extraction kit (Calbiochem, La Jolla, CA) according to the manufacturer's protocol. Briefly, after treatment, neurons were harvested and washed twice with wash buffer and were suspended in 50 *μ*L extraction buffer I containing protease inhibitor cocktail and lysed by gently rocking for 5 min. Cell debris and heavy membrane organelles were pelleted by centrifugation at 10,000 rpm for 10 min. The supernatant containing cytosolic fraction was collected, and protein concentrations were quantified by BCA protein assay kit (Beyotime Institute of Biotechnology, Shanghai, China). The purity of cytosolic fractions was verified by the absence of nuclear marker Histone H3.

### 2.13. Protein Analysis by Western Blot Analysis

Equal amounts of protein (40 *μ*g per lane) were heated to 95°C for 5 min followed by storing on ice for 5 min, separated with 8% or 10% sodium dodecyl sulfate polyacrylamide gel electrophoresis (SDS-PAGE), and electrophoretically transferred to polyvinylidinene fluoride membranes (Millipore, Bedford, MA, USA) for 90 min at a constant current of 200 mA. After sealing the membranes with 5% skimmed milk at room temperature for 2 h, 1 : 500~1000 dilution of specific primary antibodies (Cell Signaling Technology, Beverly, MA, USA) against NLRP3, cleaved-caspase-1, pro-caspase-1, GSK3*β*, phosphorylated GSK3*β*, PKA, phosphorylated PKA, PP2A, phosphorylated PP2A, p62, Keap1, Nrf2, SOD, HO-1, NQO1, Tau, p-Tau, *α*-tubulin, Histone H3, or GAPDH were incubated with the membranes for overnight at 4°C. Subsequently, the membranes were washed with TBST (Tris-buffered saline containing 0.1% Tween 20) and incubated with 1 : 5000 dilution of HRP-conjugated species-specific secondary antibody (Santa Cruz Biotechnology, Santa Cruz, CA, USA) at room temperature for 2 h. The immunoreactive bands were detected with Amersham ECL plus Western blotting detection reagent (Santa Cruz Biotechnology, Santa Cruz, CA, USA), and images were scanned and recorded with Odyssey System (LI-COR Biosciences, Lincoln, NE, USA). The gray values of protein bands were analyzed with Image J v1.8.0 software. The values of GAPDH served as internal control for whole cell or cytosolic proteins, and values of Histone H3 served as an internal control for nuclear proteins. During the examination of nuclear proteins, *α*-tubulin was used to rule out contamination of cytosolic component. The relative expression of target protein was calculated according to the equation: gray value of target protein band/gray value of control protein band.

### 2.14. Transient Transfection

Small interfering RNA (siRNA) and plasmid were transiently transfected with lipofectamine RNAiMAX transfection reagent (Thermo Fisher Scientific, Waltham, USA). siRNA against mouse p62 (5′-CGAGGAATTGACAATGGCCAT-3′) and scramble control siRNA (5′-UUCUCCGAACGUGUCACGUTT-3′) were purchased from Cell Signaling Technology (Beverly, MA, USA), and Keap1 overexpression plasmid (5′-AGTGGCGAATGATCACAGCAAT-3′) and random control plasmid (5′-ACGUGACACGUUCGGAGAATT-3′) were designed and constructed by GenePharma (Shanghai, China). Briefly, on reaching 50% confluency, 10 *μ*L lipofectamine and 5 *μ*g siRNA/plasmid were mixed for 20 min, followed by incubation with neurons for 6 h at 37°C. Thereafter, neurons were washed with PBS and cultured in culture media for 48 h and exposed to respective treatment. The transfection efficiency was examined via Western blot analysis 48 h after transfection.

### 2.15. Statistical Analysis

Data were presented as mean values with standard deviations. All experiments were performed with 5 independent repeats carried out in different cultures. Group differences were assessed with paired two-tailed Student's *t*-test or one-way ANOVA, followed by post hoc Tukey testing. All analyses were performed using SPSS version 13.0, and *p* ≤ 0.05 was considered 95% confidence limits as a significant difference.

## 3. Results

### 3.1. TNF-*α* Induced p-Tau Accumulation in Hippocampal Neurons in a Concentration- and Time-Related Manner

**Figure 1:**
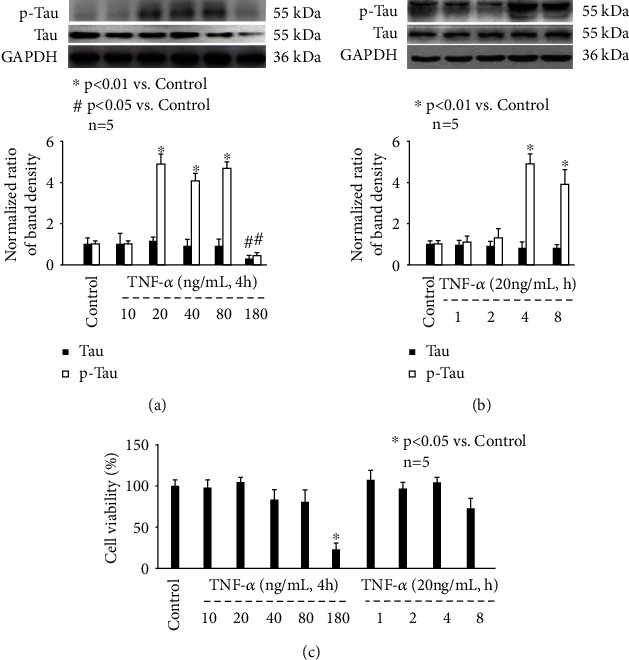
The effect of TNF-*α* on p-Tau accumulation and cell viability in mouse hippocampal neurons. (a) TNF-*α* treatment for 4 h induced p-Tau in a concentration-dependent manner. The upper panel was a representative experiment, and the lower panel was the summary of densitometric data from 5 separate experiments. GAPDH served as loading control. Data were expressed as normalized ratio of protein band density of Tau or p-Tau against GAPDH and were presented as mean ± standard deviation. (b) 20 ng/mL TNF-*α* induced p-Tau in a time-dependent manner. (c) The effect of TNF-*α* treatment on cell viability. Data were expressed as the percentage of absorbance of treated neurons compared with control neurons.

To mimic *in vivo* neuroinflammation, we treated primary mouse hippocampal neurons with different concentrations of inflammation mediator TNF-*α* (10, 20, 40, 80, and 160 ng/mL) for 4 h. By Western blot analysis, we found that TNF-*α* (10~80 ng/mL) had minor effect on Tau protein expression, which was reduced by 160 ng/mL TNF-*α* (Figure 1(a)). In addition, we found that 20~80 ng/mL TNF-*α* induced the amount of p-Tau, which was also inhibited by 160 ng/mL TNF-*α* (Figure 1(a)). Next, we treated neurons with 20 ng/mL TNF-*α* for different durations (1, 2, 4, and 8 h), and showed that although TNF-*α* (1~8 h) had no effect on Tau protein expression, 4- and 8-h treatment induced its phosphorylation status (Figure 1(b)). We also examined the effect of TNF-*α* on neuron viability and showed that 10, 20, 40, and 80 ng/mL TNF-*α* treatment for 4 h had minor effect on neuron viability, which was greatly reduced by 160 ng/mL TNF-*α* (Figure 1(c)). In addition, we showed that treatment of neurons with 20 ng/mL TNF-*α* for different durations (1, 2, 4, and 8 h) did not affect cell viability (Figure 1(c)). We postulated the reduced expression and phosphorylation of Tau after 160 ng/mL TNF-*α* exposure was due to suppress cell viability. Thereafter, 20 ng/mL TNF-*α* incubation for 4 h was considered an optimal stimulus to induce p-Tau without affecting neuron viability and was applied in the following experiments.

### 3.2. Propofol Pretreatment Concentration Dependently Prevented TNF-*α*-Induced p-Tau Accumulation in Hippocampal Neurons

**Figure 2:**
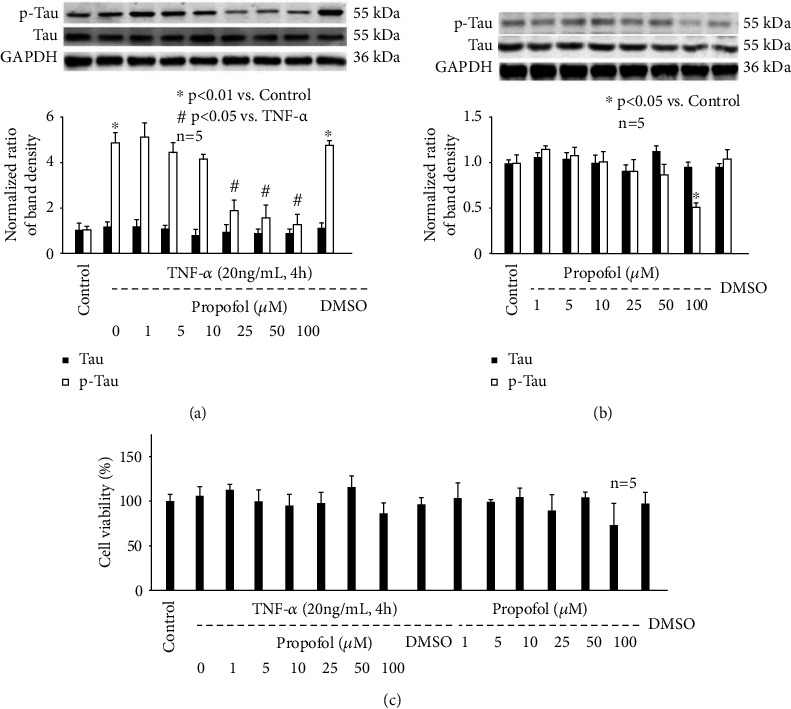
The effect of propofol on p-Tau accumulation and cell viability in mouse hippocampal neurons. (a) Propofol reduced p-Tau in hippocampal neurons exposed to TNF-*α* (20 ng/mL, 4 h). The upper panel was a representative experiment, and the lower panel was the summary of densitometric data from 5 separate experiments. GAPDH served as loading control. Data were expressed as normalized ratio of protein band density of Tau or p-Tau against GAPDH and were presented as mean ± standard deviation. (b) The effect of propofol on p-Tau in untreated hippocampal neurons. (c) The effect of propofol on neuron viability. Data were expressed as the percentage of absorbance of treated neurons compared with untreated control neurons.

To mimic clinical administration of propofol, and to exam the effect of propofol on p-Tau accumulation, we incubated hippocampal neurons with different concentrations (1, 5, 10, 25, 50, and 100 *μ*M) of propofol or 0.1% DMSO for 1 h, followed by TNF-*α* treatment (20 ng/mL, 4 h). Please note that this concentration range covers plasma concentrations of propofol during general anesthesia and sedation in clinical practice. As shown in Figure 2(a), we indicate that both propofol and DMSO had no effect on Tau protein expression. However, 25, 50, and 100 *μ*M propofol attenuated TNF-*α*-induced p-Tau accumulation, which was not affected by DMSO. In addition, we observed the effect of propofol or DMSO alone in neurons and revealed they had no effect on basal status of Tau protein expression (Figure 2(b)). Interestingly, we detected 100 *μ*M propofol reduced p-Tau accumulation, while DMSO had no such effect (Figure 2(b)). We also reported that neither propofol nor DMSO affected neuron viability (Figure 2(c)). Accordingly, we believed that the effect of propofol was independent of its solvent DMSO and thereafter further investigated detailed mechanisms for 25 and 100 *μ*M propofol-regulated p-Tau accumulation in hippocampal neurons.

### 3.3. The Effect of TNF-*α* and Propofol on Mitophagy, ROS, NLRP3 Inflammasome, and GSK3*β*/PP2A in Hippocampal Neurons

**Figure 3:**
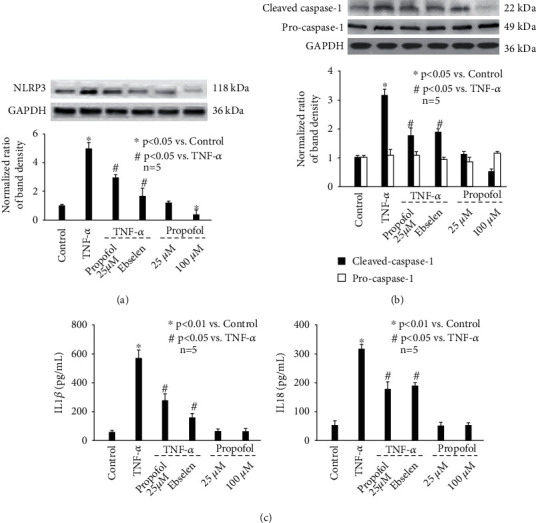
The effect of TNF-*α* and propofol on NLRP3 inflammasome activation. (a) The effect of TNF-*α* and propofol on NLRP3 protein expression. The upper panel was a representative experiment, and the lower panel was the summary of densitometric data from 5 separate experiments. GAPDH served as loading control. Data were expressed as normalized ratio of protein band density of NLRP3 against GAPDH and were presented as mean ± standard deviation. (b) The effect of TNF-*α* and propofol on pro-caspase-1 cleavage. (c) The effect of TNF-*α* and propofol on the release of matured IL-l*β* (left) and IL-18 (right). Data were expressed as mean ± standard deviation, and pg/mL served as unit.

**Figure 4:**
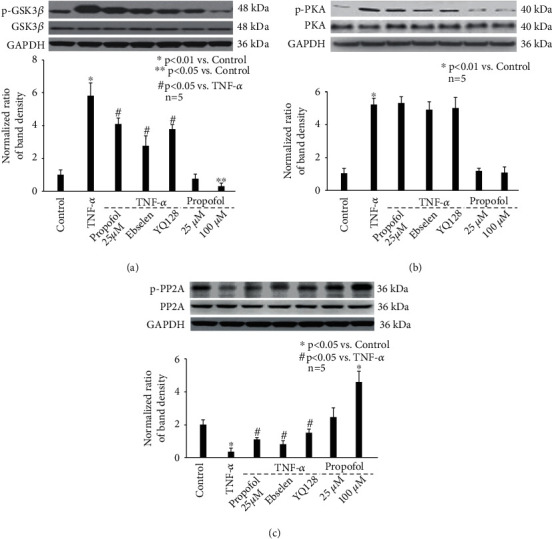
The effect of TNF-*α* and propofol on kinase/phosphatase phosphorylation. (a) The effect of TNF-*α* and propofol on the expression and phosphorylation of GSK3*β*. The upper panel was a representative experiment and the lower panel was the summary of densitometric data from 5 separate experiments. GAPDH served as loading control. Data were expressed as normalized ratio of protein band density of phosphorylated GSK3*β* against GSK3*β*, which was normalized with GAPDH, and were presented as mean ± standard deviation. (b) The effect of TNF-*α* and propofol on the expression and phosphorylation of PKA. (c) The effect of TNF-*α* and propofol on the expression and phosphorylation of PP2A.

Recently, a large body of evidence implied the correlation between NLRP3 inflammasome activation and Tau phosphorylation, which relies on the balance between kinases (GSK3*β* and PKA) and phosphatase (PP2A) activity [22–24]. As such, we examined the effect of TNF-*α* and propofol on the activation of NLRP3 inflammasome, GSK3*β*, PKA, and PP2A. In this *in vitro* study, NLRP3 inflammasome activation was evaluated by measuring NLRP3 expression, cleavage of pro-caspase-1, and release of matured IL-l*β* and IL-18. As shown in Figure 3, we demonstrate that TNF-*α* (20 ng/mL, 4 h) increased NLRP3 expression (Figure 3(a)), induced the cleavage of pro-caspase-1 (Figure 3(b)), and increased matured IL-l*β* and IL-18 release (Figure 3(c)), which were all attenuated by 25 *μ*M propofol pretreatment (Figure 3). Besides, we identified that TNF-*α* (20 ng/mL, 4 h) increased the phosphorylation of GSK3*β* (Figure 4(a)) and PKA (Figure 4(b)), while reduced PP2A phosphorylation (Figure 4(c)). Although 25 *μ*M propofol did not affect TNF-*α*-modulated PKA phosphorylation (Figure 4(b)), it ameliorated GSK3*β* phosphorylation and increased PP2A phosphorylation (Figures 4(a) and 4(c)). In addition, compared with control neurons, 25 *μ*M propofol alone did not affect NLRP3 expression, pro-caspase-1 cleavage, or matured IL-l*β*/IL-18 release, while 100 *μ*M propofol marked reduced NLRP3 expression and pro-caspase-1 cleavage (Figures 3(a) and 3(b)). In consistence, 25 *μ*M propofol alone had no significant effect on the phosphorylation of GSK3*β*, PKA, or PP2A; however, 100 *μ*M propofol decreased GSK3*β* phosphorylation and increased PP2A phosphorylation (Figure 4).

**Figure 5:**
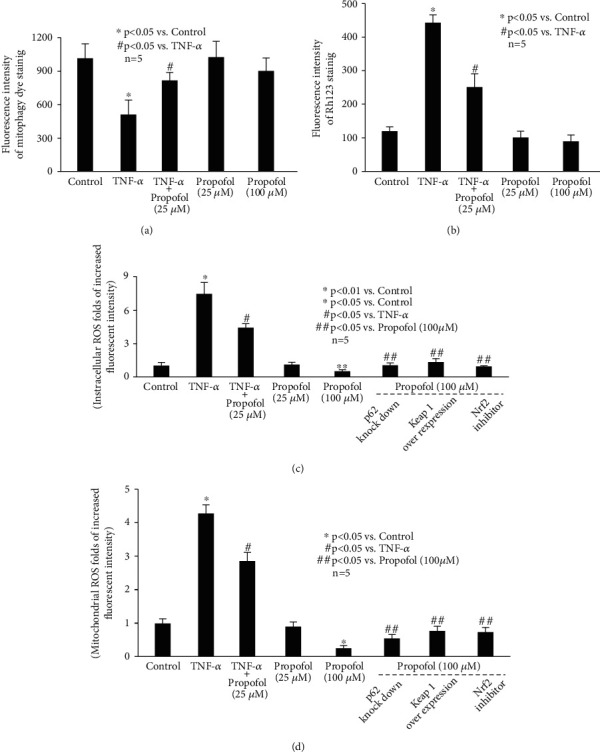
The effect of TNF-*α* and propofol on mitophagy and ROS. (a) The effect of TNF-*α* and propofol on the extent of mitophagy. Data were expressed as mean ± standard deviation of fluorescence intensity of mitophagy dye staining. (b) The effect of TNF-*α* and propofol on MMP values. Data were expressed as mean ± standard deviation of fluorescence intensity of Rh123 staining. (c) The effect of TNF-*α* and propofol on intracellular ROS. p62 siRNA, Keap1 overexpression plasmid, and Nrf2 inhibitor were used to modulate p62/Keap1/Nrf2 pathway. The data were recorded as folds of increased fluorescence intensity in treated neurons compared with that of untreated neurons. (d) The effect of TNF-*α* and propofol on mitochondrial ROS. Data were recorded as folds of increased fluorescence intensity in treated neurons compared with that of untreated neurons.

Furthermore, it has been suggested that abnormal mitophagy and resultant ROS dysregulation serve as an important mediator for NLRP3 inflammasome activation [22–24]. So, we examined the effect of TNF-*α* and propofol on mitophagy and ROS balance. As shown in Figure 5, we show that TNF-*α* (20 ng/mL, 4 h) inhibited the extent of mitophagy (Figure 5(a)), disrupted MMP values (Figure 5(b)), and induced intracellular ROS (Figure 5(c)) as well as mitochondrial ROS (Figure 5(d)). And all these effects were ameliorated by 25 *μ*M propofol pretreatment (Figure 5). Please note that 25 *μ*M propofol alone did not affect mitophagy or ROS. While interestingly, we discovered that compared with control neurons, 100 *μ*M propofol had no effect on mitophagy (Figures 5(a) and 5(b)), but it reduced intracellular ROS (Figure 5(c)) and mitochondrial ROS (Figure 5(d)) to a lower extent than the basic levels.

**Figure 6:**
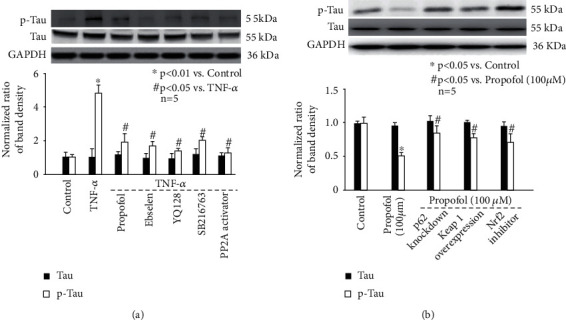
The effect of specific signal modulators on p-Tau accumulation in hippocampal neurons. The upper panel was a representative experiment and the lower panel was the summary of densitometric data from 5 separate experiments. GAPDH served as loading control. Data were expressed as normalized ratio of protein band density of Tau or p-Tau against GAPDH and were presented as mean ± standard deviation. (a) The effect of TNF-*α*, propofol, ROS scavenger, NLRP3 inhibitor, GSK3*β* inhibitor, and PP2A activator. (b) The effect of propofol and p62/Keap1/Nrf2 pathway regulators: p62 siRNA, Keap1 overexpression plasmid, and Nrf2 inhibitor.

To confirm the role of ROS, NLRP3 inflammasome, GSK3*β*, and PP2A in modulating p-Tau accumulation, we pretreated neurons with specific inhibitors or activators. As shown in Figure 6(a), TNF-*α*-induced p-Tau accumulation is attenuated by 40 *μ*M ebselen (a ROS scavenger), 1 *μ*M YQ128 (a NLRP3 inhibitor), 10 *μ*M SB216763 (a GSK3*β* inhibitor), and 1 *μ*M PP2A activator. In addition, we revealed that 40 *μ*M ebselen inhibited TNF-*α*-induced activation of NLRP3 inflammasome (Figure 3). It also ameliorated TNF-*α*-mediated phosphorylation of GSK3β (Figure 4(a)) and PP2A (Figure 4(c)). Consistently, 1 *μ*M YQ128 reduced GSK3*β* phosphorylation (Figure 4(a)), while induced PP2A phosphorylation (Figure 4(c)). Summarized from above data, we believed ROS functioned upstream of NLRP3 inflammasome, which modulates GSK3*β* and PP2A phosphorylation, leading to p-Tau accumulation.

### 3.4. The Effect and Mechanism of Propofol on Antioxidant Enzyme Expression

**Figure 7:**
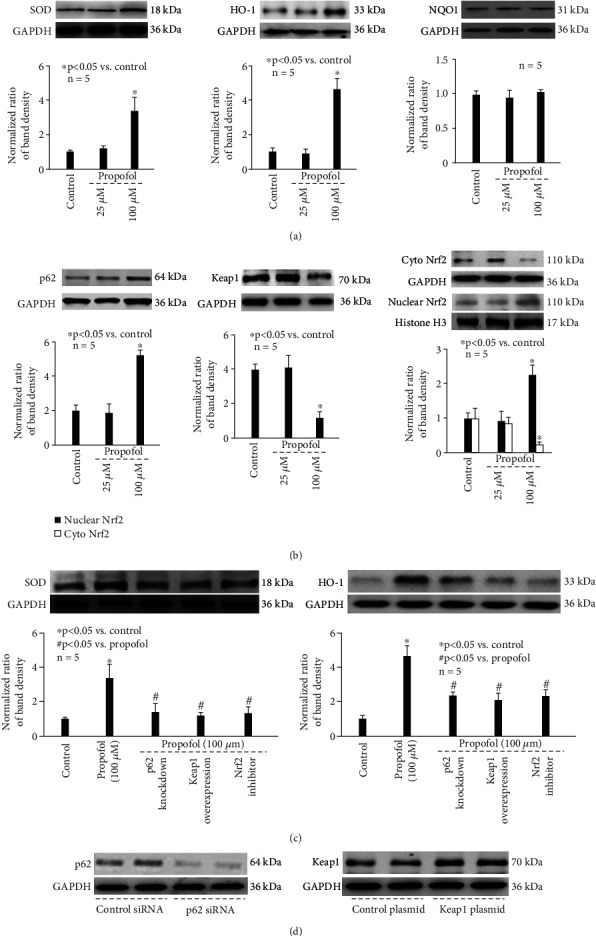
The effect and mechanism of propofol on antioxidant enzyme expression. (a) Left: SOD; middle: HO-1; and right: NQO1. The upper panel was a representative experiment, and the lower panel was the summary of densitometric data from 5 separate experiments. GAPDH served as loading control. Data were expressed as normalized ratio of protein band density of SOD, HO-1, or NQO1 against GAPDH and were presented as mean ± standard deviation. (b) Left: p62; middle: Keap1; and right: Nrf2. The upper panel was a representative experiment, and the lower panel was the summary of densitometric data from 5 separate experiments. GAPDH served as loading control for p62, Keap1, and cytosolic Nrf2. Histone H3 served as loading control for nuclear Nrf2. Data were expressed as normalized ratio of protein band density of p62, Keap1, or Nrf2 against loading control and were presented as mean ± standard deviation. (c) The effect of p62/Keap1/Nrf2 pathway regulators on antioxidant enzyme expression. Left: SOD; middle: HO-1; and right: NQO1. The upper panel was a representative experiment, and the lower panel was the summary of densitometric data from 5 separate experiments. GAPDH served as loading control. (d) The siRNA and plasmid transfection efficiency. Left: a representative immunostaining of duplicate p62 siRNA and duplicate scramble siRNA transfection. Right: a representative immunostaining of duplicate Keap1 plasmid and duplicate control plasmid transfection.

As shown in Figure 2(b), we report that compared with untreated neurons, 100 *μ*M propofol reduced basic levels of p-Tau. In addition, 100 *μ*M propofol reduced ROS (Figures 5(c) and 5(d)) without affecting the extent of mitophagy (Figures 5(a) and 5(b)). The underlying mechanism of how ROS was reduced was of great interest. It is known that cellular ROS homeostasis is modulated by their synthesis and their scavenging through the antioxidant machinery with SOD, HO-1, and NQO1 acting as major antioxidants in CNS [25]. We found that 25 *μ*M propofol alone had no effect on the expression of SOD and HO-1, which was markedly induced by 100 *μ*M propofol (Figure 7(a) left and 7a middle). In contrast, 25 *μ*M and 100 *μ*M propofol had no effect on NQO1 expression (Figure 7(a) right).

Previous findings implied p62/Keap1/Nrf2 pathway as a key mechanism for SOD and HO-1 expression [26, 27]. Our data demonstrated that 25 *μ*M propofol did not affect the expression of p62 (Figure 7(b) left) or Keap1 (Figure 7(b) middle), and did not affect the nuclear translocation of Nrf2 (Figure 7(b) right). However, 100 *μ*M propofol induced p62 (Figure 7(b) left), reduced Keap1 expression (Figure 7(b) middle), and triggered the nuclear translocation of Nrf2 (Figure 7(b) right). More importantly, we found that 100 *μ*M propofol-induced SOD and HO-1 expression was mitigated by blocking p62 expression through p62 siRNA, by enhancing Keap1 expression through Keap1 overexpression, and by 10 *μ*M ML385 (Nrf2 inhibitor) treatment (Figure 7(c)), suggesting the critical role of p62, Keap1 and Nrf2. Consistently, 100 *μ*M propofol-modulated ROS and p-Tau accumulation was abolished by p62 knockdown, Keap1 overexpression, and ML385 treatment (Figures 5(c) and 5(d) and 6(b)). The efficiency of p62 knockdown and Keap1 overexpression was demonstrated by immunostaining (Figure 7(d)).

## 4. Discussion

In the present study, we investigated the effect and mechanism of inflammation mediator TNF-*α* and anesthetic agent propofol on p-Tau accumulation in mouse hippocampal neurons. Our data implied that TNF-*α* may induce p-Tau accumulation via inhibiting mitophagy, inducing ROS, which modulated NLRP3 and GSK3*β*/PP2A activity. More meaningfully, we proved that propofol may inhibit p-Tau accumulation through modulating mitophagy, ROS, and related events, and through enhancing SOD and HO-1 expression via p62/Keap1/Nrf2 signal pathway.

Tau is a microtubule-associated protein that is predominantly expressed in the brain. In healthy neurons, Tau is almost exclusively located in the axon and is closely associated with the proper functioning of the cytoskeletal network in terms of microtubule assembly. Under normal conditions, Tau contributes to maintain neuronal functions such as transport of mRNA and proteins along the axons, microtubule stabilization, actin reorganization, and synaptic activity as well as neurite extension. In contrast, Tau pathology may cause neurofibrillary tangles and neuronal dysfunction, which are closely correlated with neurodegenerative disorders [5, 28]. Besides protein expression level, posttranslational modifications of Tau, such as phosphorylation, nitration, ubiquitination, truncation, glycosylation, and isomerization, are proved to influence its function [28]. During the past decades, increasing evidence indicated that abnormally phosphorylated Tau plays a critical role during the development of AD in animal studies and clinical trials [9, 21, 28]. Although systematic inflammation and neuroinflammation are widely accepted as a central process to the pathogenesis of neurodegenerative disorders [1–3], detailed mechanism is far from clear. Here, in the present, *in vitro* study, we treated mouse hippocampal neurons with inflammation mediator TNF-*α* to mimic *in vivo* neuroinflammation status and proved that TNF-*α* may induce the accumulation of p-Tau in neurons (Figure 1(a)). Our data at least provide a potential linkage between inflammation and neuron dysfunction, and this needs to be further studied in animal models. In addition, we reported that TNF-*α*-induced accumulation of p-Tau was attenuated by propofol pretreatment (Figure 2(a)), and one of the astonishing findings of our study is that propofol, within clinically achieved concentrations, may reduce the basic level of p-Tau (Figure 2(b)). Although whether propofol exerts beneficial or detrimental effects to CNS in the clinical practice is debatable [29–31], a large body of *in vitro* evidence from us and other researchers proved the anti-inflammation and neuro-protective property of propofol [16–19]. The current findings implied a novel research field to the neuro-protective effect of propofol and more importantly provided a novel target for the protection of neurons and neurodegenerative disorders against inflammation.

The phosphorylation status of Tau relies on the balance between kinases (including but not limited to glycogen synthase kinase, cyclin-dependent kinase, mitogen-activated protein kinase, and microtubule affinity regulating kinase) that phosphorylate it and one major phosphatase (PP2A) that dephosphorylates it [32]. It is noted that different kinases are responsible for the hyperphosphorylation of Tau protein in response to various stimuli. For example, ischemia/reperfusion injury induced Tau phosphorylation through cyclin-dependent kinase 5 (CDK5) [33]; sleep disturbances-associated Tau phosphorylation was mediated via p38 mitogen-activated protein kinase (p38MAPK) [34]; virus infection enhanced Tau phosphorylation by double-stranded RNA-dependent protein kinase [35]; sepsis triggered Tau phosphorylation through GSK-3*β* [36]; and metal dysregulation activated rapamycin/ribosomal S6 protein kinase and thus caused Tau phosphorylation [37]. In a previous study, streptozotocin was intracranially injected into the rats to induce neuroinflammation, and it was revealed that plasma TNF-*α* release was increased and p-Tau was induced by GSK-3*β* in the hippocampus area [38]. Consistently, a recent animal study carried out in mice demonstrated chronic systemic exposure to lipopolysaccharide caused neuroinflammation by promoting TNF-*α* release and triggered Tau hyperphosphorylation through activating GSK-3*β* [39]. In addition to GSK-3*β*, the role of protein kinase A (PKA) in hippocampus Tau phosphorylation and neuroinflammation-related cognitive deficits has been confirmed in animal AD models [40, 41]. Since we focused on inflammation-induced Tau phosphorylation in the current study, we only examined GSK-3*β* and PKA and proved that both were activated by TNF-*α* (Figure 4). However, our data implied that propofol only modulated GSK-3*β* activation (Figure 4). In addition, we examined phosphatase PP2A and showed that TNF-*α* inhibited PP2A activity, which was induced by propofol (Figure 4). In consistence, we showed that TNF-*α*-induced p-Tau accumulation was attenuated by propofol, GSK-3*β* inhibitor, and PP2A activator (Figure 6). Taken together, we inferred that the effect of propofol on p-Tau accumulation was mediated through modulating kinase (GSK-3*β*) and phosphatase (PP2A) simultaneously.

Inflammasome is a type of cytosolic multiprotein complex and plays a crucial role in innate immunity. Among reported inflammasomes, NLRP3 inflammasome is the most studied. More and more experimental evidence showed that the activation of NLRP3 inflammasome is closely related to neurodegenerative diseases [22]. Recently, the role of NLRP3 inflammasome in tauopathy-induced neurodegeneration attracts extensive attention [24]. It was published that tauopathy (Tau hyperphosphorylation) and neurodegeneration (hippocampal atrophy) were decreased in the NLRP3-deficient mice compared with wild type mice, implying its critical role [42]. However, how NLRP3 inflammasome modulates Tau phosphorylation is far from clear. It was shown that GSK-3*β* activity was reduced, while PP2A activity was increased in NLRP3 knockout mice [24]. This study also revealed that the kinase activity of CDK5 and p38MAPK remained unchanged in NLRP3 knockout mice [24]. Consistently, it was recognized that GSK-3*β* and PP2A were subject to NLRP3 inflammasome activation and were responsible for regulating Tau protein phosphorylation in neuronal cells [43]. Combined with our findings that propofol inhibited TNF-*α*-modulated NLRP3 inflammasome activation and GSK-3*β*/PP2A activity (Figure 4) as well as Tau phosphorylation (Figure 6), we believed that the beneficial effects of propofol on p-Tau accumulation were via inhibiting NLRP3 inflammasome-mediated GSK-3*β*/PP2A activity.

NLRP3 inflammasome activation generally requires two steps: priming and protein complex assembly. Priming is triggered by pattern recognition receptor signals, leading to transcriptional activation of NLRP3 inflammasome components. Protein complex assembly is correlated with NLRP3 inflammasome activation, leading to inflammatory response through caspase-1 activation and inflammatory cytokine IL-1*β* maturation and secretion. Although a variety of external or host-derived stimuli, such as mitochondrial dysfunction, ion flux, and lysosomal damage are involved in the activation of NLRP3 inflammasome [43], recent studies focused on mitochondrial autophagy and subsequent oxidative stress [44–46]. It was reported that in intracerebral hemorrhage brain injury model, ROS was elevated, and NLRP3 inflammasome pathway was activated [47]. It also reported that ROS scavenger may inhibit NLRP3 inflammatory response and alleviate brain injury [47]. Another study proved during ischemia/reperfusion injury, mitochondria malfunction caused ROS accumulation and stimulated NLRP3 inflammasome activation [48]. In addition, it was shown that increased ROS, which is duo to impaired mitophagy, contributed to NLRP3 inflammasome signaling activation in neurodegenerative diseases [49, 50]. Here in this study, our data also suggested a correlation between mitophagy, ROS, and NLRP3 inflammasome activation after TNF-*α* treatment (Figures 3 and 5). Based on the findings that propofol and ROS scavenger could reduce intracellular ROS and NLRP3 inflammasome activation (Figures 3 and 5), we concluded that ROS was indispensable for NLRP3 inflammasome activation.

In general, we deduced from this *in vitro* study that TNF-*α* impaired neuron mitophagy, caused excessive oxidative stress, which activated NLRP3 inflammasome, resulting in dysregulation of GSK-3*β*/PP2A activity and advanced p-Tau accumulation. Further, we believed that propofol may decrease p-Tau accumulation via enhancing mitophagy, reducing oxidative stress and subsequent events. Nevertheless, we discovered an interesting phenomenon that relative high concentration of propofol (100 *μ*M) reduced basic level of p-Tau (Figure 2(a)). It also modulated GSK-3*β*/PP2A phosphorylation (Figure 4), NLRP3 inflammasome activity (Figure 3), and ROS (Figures 5(c) and 5(d)). However, 100 *μ*M propofol had no significant effect on mitophagy (Figures 5(a) and 5(b)). The potential mechanisms for reduced ROS in this scenario deserve investigations.

It is recognized that the cellular ROS homeostasis is modulated by their synthesis, mainly through NADPH oxidase complex, and their scavenging through the antioxidant machinery with glutathione and ascorbate acting as major antioxidants. Among multiple antioxidant enzymes, SOD, HO-1, and NQO1 were extensively studied in hippocampal neurons and neurological disorders [25, 51–54]. SOD is a group of metal-containing enzymes that catalyze the dismutation of superoxide radicals to molecular oxygen and hydrogen peroxide, providing cellular defense against reactive oxygen species. It was reported that sinomenine may improve hippocampal and cognitive dysfunction through modulating SOD activity and ROS, while it was also suggested that catalase, glutathione reductase, glutathione peroxidase, and myeloperoxidase were not involved [55]. HO-1 is a cytoprotective enzyme that catalyzes the degradation of heme to carbon monoxide, iron, and biliverdin, and its induction has been regarded as an adaptive cellular response against inflammatory response and oxidative injury. It was reported that increased expression of HO-1 was correlated with less intracellular ROS and improved neuronal and neurological function in mice [56]. NQO1 is a cytosolic enzyme which catalyzes the reduction of quinones and a wide variety of other compounds. It is often upregulated in response to cellular stress, and it has a role in minimizing free radical load within cells. Previous study showed that increased NQO-1 activation was correlated with less ROS and improved cell viability in hippocampal neuronal cells [57]. Animal study also proved that upregulated NQO-1 expression was correlated with reduced oxidative stress and improved neurological status in rats following traumatic brain injury [58]. Here in the present study, we demonstrated that SOD and HO-1 expression were induced by 100 *μ*M propofol; however, we did not detect modulation of NQO-1 (Figure 7).

Speaking of the molecular mechanisms for antioxidant enzyme activation in CNS, plenty of data pointed to p62/Keap1/Nrf2 pathway [51, 56, 58]. It is believed that p62 aggregation leads to Keap1 degradation by autophagosomes. Under normal conditions, Keap1 functions as an adapter protein of the Cul3-ubiquitin E3 ligase complex responsible for degrading Nrf2. Accordingly, increased p62 leaves Nrf2 free to transfer to the nucleus and binds to antioxidant-responsive elements in the promoter of antioxidant enzymes. We believed that p62/Keap1/Nrf2 pathway was responsible for propofol-modulated SOD/HO-1 expression and p-Tau accumulation, since p62 knockdown/Keap1 overexpression/Nrf2 inhibitor almost completely blocked the beneficial effects of propofol (Figure 7(c) and 6(b)).

We realized there are several issues that are unsolved in this study and deserve further investigations. Firstly, it is known that neuronal TNF-*α* receptor (TNFR), such as TNFR-I and TNFR-II perform fundamentally different roles in CNS pathology [59], while we did not examine which receptor is responsible for TNF-*α*-induced p-Tau accumulation in hippocampal neurons. Also, we did not investigate whether propofol affects the expression and activation of specific TNFR. The answer to these questions may reveal a novel therapeutic target for tauopathy. Secondly, in this study, in order to investigate the protective effects of propofol, its exposure was 1 h ahead of TNF-*α*. While we did not know whether propofol could reverse the accumulation of p-Tau when the neurons have already been exposed to TNF-*α* for a while, this needs to be revealed, and the results may provide solid evidence for the beneficial property of propofol in those patients who have been suffered from neuroinflammation.

## 5. Conclusions

In conclusion, we testified that TNF-*α* may induce p-Tau accumulation via inhibiting mitophagy, inducing ROS, which modulated NLRP3 and GSK3*β*/PP2A activity. We also proposed that propofol may inhibit p-Tau accumulation through modulating mitophagy, ROS, and resultant events and through enhancing SOD and HO-1 expression via p62/Keap1/Nrf2 pathway.

## Data Availability

The data that support the findings of this study are available from the corresponding author upon reasonable request.
